# Cholangiocarcinoma: Recent Advances in Molecular Pathobiology and Therapeutic Approaches

**DOI:** 10.3390/cancers16040801

**Published:** 2024-02-16

**Authors:** Divya Khosla, Shagun Misra, Pek Lim Chu, Peiyong Guan, Ritambhra Nada, Rajesh Gupta, Khwanta Kaewnarin, Tun Kiat Ko, Hong Lee Heng, Vijay Kumar Srinivasalu, Rakesh Kapoor, Deepika Singh, Poramate Klanrit, Somponnat Sampattavanich, Jing Tan, Sarinya Kongpetch, Apinya Jusakul, Bin Tean Teh, Jason Yongsheng Chan, Jing Han Hong

**Affiliations:** 1 Department of Radiotherapy and Oncology, Post Graduate Institute of Medical Education and Research, Chandigarh 160012, India; 2 Department of Radiotherapy and Oncology, Sanjay Gandhi Postgraduate Institute of Medical Sciences, Lucknow 226014, India; 3 Cancer and Stem Cell Biology Programme, Duke-NUS Medical School, Singapore 169857, Singapore; 4 Genome Institute of Singapore, Agency for Science, Technology and Research (A*STAR), Singapore 138672, Singapore; 5 Department of Histopathology, Post Graduate Institute of Medical Education and Research, Chandigarh 160012, India; 6 Department of GI Surgery, HPB, and Liver Transplantation, Post Graduate Institute of Medical Education and Research, Chandigarh 160012, India; 7 SingHealth Duke-NUS Institute of Biodiversity Medicine, Singapore 168583, Singapore; 8 Cancer Discovery Hub, National Cancer Center Singapore, Singapore 168583, Singapore; 9 Laboratory of Cancer Epigenome, Division of Medical Science, National Cancer Center Singapore, Singapore 168583, Singapore; 10 Department of Medical Oncology, Mazumdar Shaw Medical Center, NH Health City Campus, Bommasandra, Bangalore 560099, India; 11 Cholangiocarcinoma Screening and Care Program (CASCAP), Khon Kaen University, Khon Kaen 40002, Thailand; 12 Department of Biochemistry, Faculty of Medicine, Khon Kaen University, Khon Kaen 40002, Thailand; 13 Siriraj Center of Research Excellence for Systems Pharmacology, Department of Pharmacology, Faculty of Medicine, Siriraj Hospital, Mahidol University, Bangkok 73170, Thailand; 14 State Key Laboratory of Oncology in South China, Collaborative Innovation Center of Cancer Medicine, Sun Yat-sen University Cancer Center, Guangzhou 510060, China; 15 Cholangiocarcinoma Research Institute, Khon Kaen University, Khon Kaen 40002, Thailand; 16 Department of Pharmacology, Faculty of Medicine, Khon Kaen University, Khon Kaen 40002, Thailand; 17 Centre for Research and Development of Medical Diagnostic Laboratories, Faculty of Associated Medical Sciences, Khon Kaen University, Khon Kaen 40002, Thailand; 18 Institute of Molecular and Cell Biology, Agency for Science, Technology and Research (A*STAR), Singapore 138673, Singapore; 19 Oncology Academic Clinical Program, Duke-NUS Medical School, Singapore 169857, Singapore; 20 Division of Medical Oncology, National Cancer Center, Singapore 168583, Singapore

**Keywords:** cholangiocarcinoma, therapeutic approaches, pathobiology

## Abstract

**Simple Summary:**

Cholangiocarcinoma (CCA) manifests as a complex interplay of genetic and environmental factors, necessitating personalized approaches. This review covers the various causes, risk factors, and molecular aspects of CCA in oncology. It explores current diagnostic methods and treatments, such as targeted therapies and immunotherapy. Additionally, it addresses emerging strategies like microbiota modulation and the use of natural compounds. This review paper provides a comprehensive understanding of CCA, offering insights into current and evolving approaches for effective interventions in oncological care.

**Abstract:**

Cholangiocarcinomas (CCA) pose a complex challenge in oncology due to diverse etiologies, necessitating tailored therapeutic approaches. This review discusses the risk factors, molecular pathology, and current therapeutic options for CCA and explores the emerging strategies encompassing targeted therapies, immunotherapy, novel compounds from natural sources, and modulation of gut microbiota. CCA are driven by an intricate landscape of genetic mutations, epigenetic dysregulation, and post-transcriptional modification, which differs based on geography (e.g., for liver fluke versus non-liver fluke-driven CCA) and exposure to environmental carcinogens (e.g., exposure to aristolochic acid). Liquid biopsy, including circulating cell-free DNA, is a potential diagnostic tool for CCA, which warrants further investigations. Currently, surgical resection is the primary curative treatment for CCA despite the technical challenges. Adjuvant chemotherapy, including cisplatin and gemcitabine, is standard for advanced, unresectable, or recurrent CCA. Second-line therapy options, such as FOLFOX (oxaliplatin and 5-FU), and the significance of radiation therapy in adjuvant, neoadjuvant, and palliative settings are also discussed. This review underscores the need for personalized therapies and demonstrates the shift towards precision medicine in CCA treatment. The development of targeted therapies, including FDA-approved drugs inhibiting *FGFR2* gene fusions and *IDH1* mutations, is of major research focus. Investigations into immune checkpoint inhibitors have also revealed potential clinical benefits, although improvements in survival remain elusive, especially across patient demographics. Novel compounds from natural sources exhibit anti-CCA activity, while microbiota dysbiosis emerges as a potential contributor to CCA progression, necessitating further exploration of their direct impact and mechanisms through in-depth research and clinical studies. In the future, extensive translational research efforts are imperative to bridge existing gaps and optimize therapeutic strategies to improve therapeutic outcomes for this complex malignancy.

## 1. Introduction

Cholangiocarcinomas (CCAs) are rare and aggressive cancers characterized by phenotypic and genotypic heterogeneity with varied epidemiology across the globe. These are classified based on anatomical location into intrahepatic (comprising 10–20%), perihilar or Klatskin tumor (50%), and distal (30–40%) CCA [1], and based on etiology into fluke-negative or fluke-positive CCA [2]. The diagnosis often poses challenges, and it is seldom diagnosed at early stages due to its silent clinical course, lack of specific tumor markers, limited sensitivity of imaging techniques as it tends to grow longitudinally along the bile duct rather than radially, and difficulty in obtaining tissue diagnosis. Despite curative resection, prognosis remains poor due to high rates of recurrence [3].

The molecular pathogenesis of CCA is complex and multifactorial. A multitude of molecular mechanisms have been implicated in the process of cholangio-carcinogenesis and may differ by anatomical location and etiology. The tumor cells undergo diverse genetic and epigenetic alterations resulting in enhanced proliferation signaling, dysregulation of apoptosis, angiogenesis, invasion, and stromal proliferation [4]. Prolonged biliary inflammation, cholestasis, and fibrosis incite aberrant activation of various receptors and deregulate intracellular signaling pathways. There is a need for a comprehensive understanding of the molecular mechanisms to stratify the patients using validated biomarkers for personalized treatment strategies.

The complex tumor microenvironment comprising a diverse array of tumor cells, highly invasive behavior, desmoplastic and hypovascular stroma, mutational landscape, development of therapeutic resistance, and intra and inter-tumoral heterogeneity has led to refractoriness to chemotherapy. Nearly half of the CCAs have targetable mutations [5,6,7]. Advances in pathology, molecular biology, and genetics have led to an improved understanding of the molecular mechanisms underlying CCA development. This enables the identification of specific molecular targets and prediction of treatment responses for more efficacious interventions. This knowledge is crucial for optimizing drug development and enhancing our overall comprehension of the biological processes underlying CCA’s development and progression.

This review will summarize the risk factors, current evidence about the pathophysiology and molecular genetics, along with potential role of liquid biopsy in diagnostics. It will also highlight the current and emerging treatment options, advances in the field of personalized medicine, the role of novel compounds from natural sources, and the therapeutic implications of modulation of gut microbiota to reverse microbial dysbiosis. Through our comprehensive summary of the molecular mechanisms underlying CCA and the recent therapeutic advances in this field, our review aims to contribute to a better understanding of CCA and to highlight existing and upcoming potential therapeutic options for patients with CCA.

## 2. Risk Factors

The global incidence of CCA has increased over the past decades and varies across distinct geographical areas, reflecting unique population risk factors and the evolving etiopathogenesis landscape. The complexity of the matter is further accentuated if dissected by intrahepatic and extrahepatic CCA subtypes, where the former has been exhibiting a rising incidence rate in European and American populations [8]. Conventional risk factors common to all CCA subtypes converge upon chronic biliary inflammation and bile stasis as the underlying pathobiological mechanism [9,10], although the specific triggers differ depending on geographical location and predisposing environmental conditions. The risk factors for different geographical locations are summarized in Table 1.

### 2.1. Risk Factors in East Asian Countries

In East Asia, the most prevalent risk factor for the development of CCA is infection with endemic parasitic liver flukes *Opisthorchis viverrini* (Ov) or *Clonorchis sinensis*, both of which are classified as Group 1 biological carcinogens by the International Agency Research on Cancer (IARC) in 2012 [11].

Infestation of liver flukes called *Opisthorchis viverrini* (Ov) has been associated with the highest incidence of CCA in the northeast region of Thailand and neighboring Laos and Cambodia [12,13]. Ov is a foodborne trematode that encysts as a metacercaria in the cyprinoid fish. Fluke infestation occurs secondary to eating raw or uncooked fish containing metacercarial cysts, which are a highly infective stage. After ingestion, the juvenile flukes migrate through the ampulla of Vater into the common bile duct and into the intrahepatic bile ducts. The metacercaria will form adult Ov, which can inhabit the biliary tract within the human host for over 10 years [14]. OV has been suggested to induce cholangiocarcinoma (CCA) through three mechanisms: (1) physical damage to biliary epithelia caused by the parasites’ feeding activities, (2) immunopathology resulting from infection-related inflammation, and (3) the deleterious effect of excretory/secretory molecules released by the parasites [15]. Ov infection promotes the activation of inflammatory cells by proinflammatory cytokines and nitric oxide, produced via inducible nitric oxide synthase. Nitric oxide, generated in infected and inflamed tissues, is implicated in CCA development by causing DNA and protein damage [16,17], leading to gene mutations. It also stimulates cyclooxygenase-2 expression, promoting cholangiocyte growth through the activation of growth factors like the epidermal growth factor receptor, mitogen-activated protein kinases, and interleukin-6 [18]. Consequently, chronic Ov infection and inflammation contribute to the accumulation of genetic, epigenetic, and transcriptional alterations in CCA.

### 2.2. Risk Factors in Western Countries

On the other hand, in the West, primary sclerosing cholangitis (PSC), a rare immune-related condition, is one of the major known risk factors for CCA. Characterized by chronic biliary inflammation leading to biliary strictures and cholestasis, PSC confers a 400-fold risk of CCA compared to the general population, resulting in an annual risk of 2% and a 30-year cumulative incidence of 20% [19,20]. Choledochal cysts, rare congenital anomalies resulting in cystic dilatation of the biliary tree, represent another well-established risk factor for CCA [21,22]. The overall risk of CCA appears to be even higher in Caroli disease and Caroli syndrome, both genetic conditions defined by cystic dilation of intrahepatic bile ducts and referred to in the Todani classification as Type 5 cysts. In a recent multicentre retrospective study across 17 hepatobiliary centers in Germany, the rate of CCA in patients with Caroli disease and Caroli syndrome was 7.1%, matching closely to previous case series and reports [23].

Liver cirrhosis, as well as cholelithiasis and choledocholithiasis, have been identified as strong risk factors for CCA, particularly intrahepatic and extrahepatic CCA subtypes, respectively [9,24]. Chronic viral infections with hepatitis B virus (HBV) and hepatitis C virus (HCV) may also represent a risk factor for CCA development, with a stronger predilection for intrahepatic CCA [25]. Notably, the increased risk of CCA amongst HBV and HCV patients not only depends on the presence of liver cirrhosis but also on the direct oncogenic effects exerted by these viruses on the liver [26]. A positive association between non-alcoholic fatty liver disease (NAFLD) and CCA has also been suggested, especially for intrahepatic CCA [27]. In addition, type 2 diabetes mellitus has been reported to confer a higher risk for CCA, whilst treatment with metformin, in turn, confers a protective role [28]. Other risk factors include inflammatory bowel disease, alcohol consumption, and tobacco smoking, while obesity and hypertension remain controversial [9].

Despite the available evidence providing support for several conditions predisposing to CCA, most patients with CCA have no identifiable risk factors, implying the presence of hitherto-undefined etiologies to be confirmed.

### 2.3. Environmental Carcinogens as Risk Factors

Previously, thorotrast, a radiographic contrast agent now banned from clinical use, has been strongly suggested to increase the risk of CCA development [29]. Interestingly, a link between asbestos exposure and CCA has also been provided in two different case-control studies. In an Italian study, occupational exposure to asbestos was significantly greater in patients with intrahepatic CCA, but not with extrahepatic CCA. These findings were similarly demonstrated in a population-based case-control study nested on the Nordic Occupational Cancer cohort, where an increased risk of intrahepatic CCA, but not of extrahepatic CCA, was observed by cumulative exposure to asbestos [30,31].

The use of traditional and complementary medicines is in an increasing trend globally. Appealing to the empirical evidence on safety and effectiveness, the World Health Organization estimated that approximately 80% of the population may rely on these medicines for their primary health care needs [32]. Aristolochic acid (AA) is a toxic compound found naturally in plants of genera Aristolochia and Asarum [33]. For more than 2500 years, AA-containing plants have been used in traditional herbal medicines and dietary supplements for their anti-inflammatory and analgesic properties. However, in the early 1990s, the first description of disease related to AA was reported in Belgium—nephropathy incidence characterized by chronic tubulointerstitial fibrosis and accompanied by a high risk of upper tract urothelial carcinoma (UTUC) [34].

AA (and plants containing it) are classified as a Group I carcinogen by the IARC. It is known that exposure to specific mutagens leads to characteristic combinations of types of somatic mutations in the genome, called mutational signatures. When AA enters cells, the metabolically activated AA binds covalently to dA and dG purine residues in DNA to form aristolactam-DNA (AL-DNA) adducts, which block DNA replication. These DNA adducts are repaired by transcription-coupled nucleotide excision repair (TC-NER) but are resistant to global genome nucleotide excision repair (GG-NER), thus leading to a marked non-transcribed strand bias with enriched A:T to T:A transversion events [35,36]. This unique mutational profile is referred to as signature 22 or single-base substitution 22 (SBS22) in the Catalogue of Somatic Mutations in Cancer (COSMIC). Strikingly, genomic profiling of UTUC and hepatocellular carcinoma (HCC) samples from Taiwan, the Balkans, and Belgium have consistently revealed an enriched SBS22 signature (AA-UTUC), including the well-known tumor suppressor gene TP53, implicating the strong mutagenic effect by AA [36,37,38].

Recent genomic profiling has demonstrated the pervasiveness of the AA mutational signature in intrahepatic and perihilar CCA, suggesting that AA might be a new exogenous risk factor for CCA development [39,40,41]. From the whole-exome sequencing of 102 iCCA patients, Zou and co-workers [39] observed a prevalence of marked strand-biased SBS22 in the CTG sequence context, which is consistent with the aforementioned AA-UTUC. Interestingly, the SBS22 mutation was positively correlated to the patients associated with liver inflammation or fibrosis alone (*p* < 0.011) or cirrhosis alone (*p* < 0.001) compared to those having none of these pathological features. Moreover, significantly higher SBS22 mutations were found in the group of patients with liver inflammation, fibrosis, or cirrhosis (*p* < 0.001). In a separate study by Lin et al. [41], 35.8% of the intrahepatic CCA patients had a SBS22 mutational signature and presented with a significantly higher tumor mutational burden (TMB) than the non-AA intrahepatic CCA patients (*p* < 0.001). An elevated TMB was found to correlate with a better response rate to the immune checkpoint blockade treatment; however, this remains to be determined for AA-CCA [42]. Nonetheless, this speculation is further consolidated by the finding of significantly increased infiltration of activated natural killer (NK) cells and enhanced CD160 expression (a marker for cytotoxic NK cell) in intrahepatic CCA tumors harboring the SBS22 signature and with a high TMB and neoantigen load [43]. Tumors harboring aflatoxin signatures were locally immunosuppressed without enrichment of immune cells or immune checkpoints, despite the higher TMB and neoantigen load observed in these tumors. The tumor-promoting effect of AA in CCA was further supported by a mouse study that investigated the mutational features of AA [44]. Exposure to AA alone or in combination with CCl4 (a hepatotoxic agent) induced the development of HCC, or combined HCC and iCCA, in a dose-dependent manner. These tumors recapitulated human AA-cancers by showing remarkably high A:T to T:A transversions on the nontranscribed strand, predominantly in the sequence context of CTG > CAG. In addition, clonality analysis demonstrated the highest variant allele frequency of A > T transversion mutations in the earliest founder clones, which were eliminated in the subsequent subclones during malignancy. Moreover, AA-driven recurrent tumors could arise from multiple independent origins, as compared to non-AA recurrent tumors that shared the clonal origin, thus inferring the complexity and tumor heterogeneity attributed to AA. Collectively, understanding the role of AA in the etiology of CCA is crucial to providing new insights into the carcinogenesis mechanisms that could refine the treatment strategies for AA-CCA.

## 3. Molecular Pathology

### 3.1. Pathological Features

CCA arises from biliary ducts. Since biliary ducts span from the canal of Hering to the common bile duct opening at the ampulla of Vater, the first step is to identify their anatomic location and growth patterns, followed by microscopic assessment for differentiation and subtype. This should be further supported by immunohistochemistry followed by background pathology to ascertain etiology and finally molecular subtyping.

### 3.2. Cholangiocarcinoma Nomenclature according to Location in Biliary Tract Anatomy

Cholangiocarcinomas have three distinct anatomical categories depending on the site of the biliary tract from where it arises. The biliary tree has intrahepatic and extrahepatic components; hence, these tumors are also classified as intrahepatic and extrahepatic CCA. An extrahepatic biliary tree has two parts, termed perihilar (pCCA) and distal cholangiocarcinoma (dCCA). dCCA includes tumors between the origin of the cystic duct from the CBD and ampulla of Vater. Perihilar CCA includes segmental ducts (second-order bile ducts) and right and left hepatic ducts, their confluence upto the insertion of the cystic duct to form a common bile duct. Intrahepatic CCA includes tumors arising from the canal of Herring, bile ductules (20 μm), interlobular bile ducts (20–100 μm), septal (>100 μm and <300 μm) first-order branches. It is important to subcategorize, as each of these categories differs in their risk factors, epidemiological features, clinical presentations, and morphologic and molecular characteristics [45,46].

### 3.3. Growth Pattern

**Figure 1:**
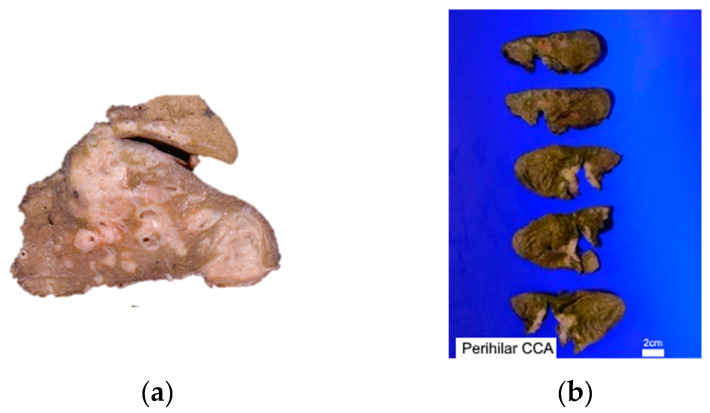
Gross photograph of (**a**) Intrahepatic CCA’s both mass-forming lesions and periductal infiltrative, (**b**) perihilar CCA-affected both right and left hepatic ducts.

After deciding the location, the growth pattern of the tumor needs to be identified. The growth patterns of intrahepatic CCA can be mass forming lesions (60–80%), periductal infiltrating (15–35%), or intraductal growth types (8–29%) (Figure 1).

Mass-forming lesions represent solid, non-encapsulated tumors found within the hepatic parenchyma. They exhibit a cut surface that is either white or greyish, often displaying central necrosis or scarring and well-defined borders. Evidence of intrahepatic metastases or the fusion of smaller lesions may be apparent, and the cut surface may manifest mucinous characteristics. These tumors are believed to originate from the small bile ducts within the liver. The periductal infiltrating type of intrahepatic CCA extends along the portal tracts, resulting in bile duct strictures with luminal narrowing. The intraductal growth variant of intrahepatic CCA presents as a papillary or polypoidal lesion within a dilated bile duct. Macroscopically, pCCA and dCCA share similar growth patterns. In about 80% of cases, they present as flat or ambiguously defined nodular sclerosing tumors, marked by thickening of the duct wall and widespread infiltration into surrounding structures. Moreover, they may appear as intraductal papillary tumors, signifying the malignant progression of intraductal papillary mucinous neoplasm (IPNB) [47].

### 3.4. Large and Small Duct Variants of Intrahepatic CCA

**Figure 2:**
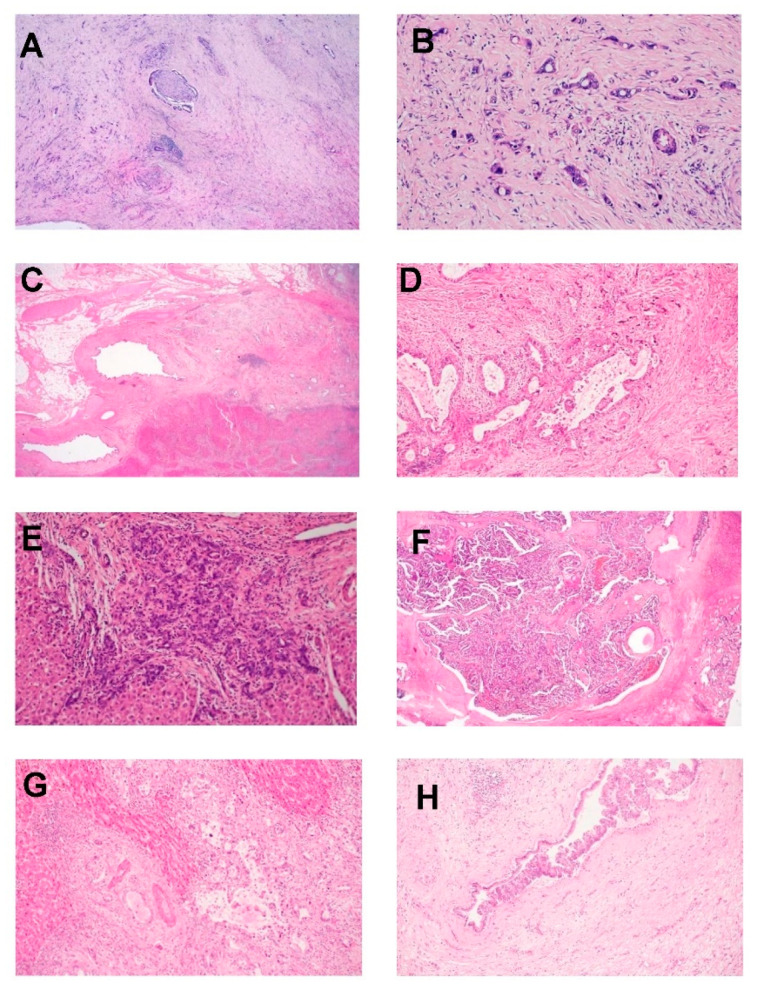
Photomicrographs show (**A**) iCCA-LD composed of glands with abundant desmoplastic stroma, perineural invasion, and portal vein thrombosis with tumor infiltration. (**B**) iCCA-LD showing malignant glands lined by cuboidal dysplastic cells having intracytoplasmic mucin; (**C**) perihilar CCA with tumor arising from the segmental intrahepatic duct, extrahepatic bile duct is normal; (**D**) tumor was composed of large glands lined by cuboidal–columnar cells with abundant intraluminal mucin; (**E**) iCCA-SD composed of small cuboidal cells without any mucin with hyperchromatic nuclei forming anastomosing cords; (**F**) perihilar CCA with an intraductal papillary configuration; (**G**) iCCA infiltration into adjacent hepatocytes; (**H**) bile duct showing evidence of biliary epithelial neoplasia-low grade with a papillary pattern that is a precursor lesion for extrahepatic CCA (H&E-A-H, 4×-A,F, ×20-B,D, ×1.25-C, ×10 F,G,H original magnification).

Intrahepatic CCA can be classified into a minimum of two primary categories based on their differentiation (see Figure 2): well/moderately-differentiated tubular/acinar adenocarcinoma, poorly-differentiated tubular/acinar adenocarcinoma, or less common morphological variations [48]. Another method of categorization involves differentiating between large and small duct types, which hold clinical, pathological, immunohistochemical, and molecular importance [47,49].

The large duct variant of intrahepatic CCA (LD-iCCA) originates from the intrahepatic bile ducts or their associated peribiliary glands. These tumors exhibit similarities to the biliary epithelium, featuring cuboidal to tall columnar cells with cytoplasmic mucin, forming extensive acini with open luminal spaces. Furthermore, they display a significant presence of desmoplastic stroma [50].

The small bile duct type of intrahepatic CCA (SD-iCCA) resembles cholangiolar cells and arises from progenitor cells or mature hepatocytes by trans-differentiation. These tumors are composed of small monotonous or anastomosing glands lined with cuboidal cells. Tumor cells have uniform nuclei with vesicular nuclei, scant to moderate eosinophilic or amphophilic cytoplasm, and no mucin production. There are no recognizable precursor lesions for SD-iCCA, whereas biliary intra-epithelial neoplasia (BIN-low and high grade) and IDPN (intraductal papillary neoplasm) are precursor lesions of LD-iCCA. Predisposing risk factors are also different for both these sub-categories; SD-iCCA are associated with chronic liver diseases/cirrhosis (especially viral hepatitis) and non-biliary cirrhosis (hemochromatosis, alcoholic liver disease, metabolic syndrome, obesity, and diabetes mellitis). LD-iCCA is associated with chronic biliary disease, precursor lesions, and hepatolithiasis. Caroli’s disease, congenital fibrosis, and bile duct cysts are predisposing factors for LD-iCCA with corresponding morphologies. SD-iCCA nearly always has a mass-forming macroscopic growth pattern. LD-iCCA has variable macroscopic growth patterns with mucin production, poorer differentiation, perineural/lymphatic invasion, and lymph node metastases [47].

Both large and small duct iCCA are stained positive with EMA (MUC1), hepatocyte nuclear factor-1β (HNF-1β), CK7, and CK 19. CK20 immunostain is typically negative or focally positive. Several studies have documented distinct immunohistochemical characteristics of LD-iCCA and SD-iCCA [51,52]. SD-iCCA are positive with NCAM (CD56), C-reactive protein, N-cadherin, and IDH1/2. LD-iCCA are positive with MUC-5AC, MUC 6, S-100, TFF1, MMP, and KRAS.

It is imperative to distinguish intrahepatic CCA from metastases originating from colorectal carcinoma, upper gastrointestinal tract malignancy, or tumors with a pancreatobiliary origin. Generally, intrahepatic CCA displays a lack of reactivity for CDX2 and SAT-B2, although there are instances where mild focal positivity for these markers may be observed. The immunostains CDX2 and SAT-B2 are instrumental in negating a diagnosis of metastatic colorectal adenocarcinoma, given its typical strong positivity for CK20, CDX2, and SAT-B2 but negativity for CK7 and CK19.

The challenge lies in differentiating between CCA and metastatic pancreatic ductal adenocarcinoma, as well as upper gastrointestinal tract carcinomas, through immunostains, as both types of tumors typically exhibit positivity for CK7 and CK19. Fernández Moro et al. [53] proposed a comprehensive immunohistochemical panel including CK19, CK20, MUC2, MUC5AC, CA19–9, mCEA, CA125, and SMAD4 to aid in the differentiation of metastatic and pancreatobiliary adenocarcinomas.

### 3.5. Molecular Genetics

CCA encompasses a highly heterogeneous genomic mutational landscape that is associated with poor outcomes in patients [2,6,7,54,55]. In a previous study on 489 CCAs spanning 10 countries, comprehensive integrative clustering revealed 4 clusters that are defined by distinct etiologies with separate genetic, epigenetic, and clinical features [2]. In brief, clusters 1 and 2 were mostly fluke-positive CCA patients with enriched TP53 mutation, ERBB2 amplification, and elevated ERBB2 expression. A wide array of genetic alterations exists, including mutations in certain tumor suppressors such as ARID1A, SMAD4, and PTEN (Table 2), implying a diverse selective pressure that drives the pathogenesis of CCA. On the other hand, cluster 3 and 4, which were primarily comprised of fluke-negative CCA patients, were enriched in BAP1 and IDH1/2 mutations and FGFR alterations. Other than geography, the mutational landscape of CCA also differs by anatomical location. Despite the fact that oncogenic mutations in CCA, such as TP53, KRAS, IDH1, ARID1A, and CDKN2A/B are commonly found in many other cancer types, it is apparent that the alteration frequencies differ substantially between intrahepatic CCA and extrahepatic CCA (eCCA) (Table 2). Furthermore, FGFR fusions were detected only exclusively in intrahepatic CCA, and the fusions were found to be mutually exclusive with FGFR/BRAF/ERBB2/KRAS mutations [56]. CCA pathogenesis could occur due to post-transcriptional modification (PTM). In a recent integrative analysis of 348 fluke-negative CCA samples (including 87 from pCCA and 261 from intrahepatic CCA), pathway analysis of driver mutations revealed enrichment in RTK-RAS, Wnt, PI3K, cell cycle, TP53, TGF-beta, and HIPPO pathways. Further analysis indicated recurring mutations in genes participating in PTM (METTL14 and RBM10) in pCCA [40]. Functional studies demonstrated that METTL14^R298H^ mutation-mediated m6A modification disrupted the repression of the MACF1/β-catenin pathway, thus, indicating the involvement of PTM in driving the occurrence and pathogenesis of CCA.

Next, epigenetic dysregulation also orchestrates CCA pathogenesis. Driver mutations in genes related to chromatin modification and DNA methylation, such as SMARCA4, PBRM1, BAP1, ARID1A, WHSC1, DNMT3A, and EZH2 were frequently identified [2,40], implying that the mutation in these epigenetic modifiers could be the primary event preceding epigenetic dysregulation, thus regulating the transcriptome driving the CCA. Distinct patterns of DNA hypermethylation targeting either promoter CpG islands or promoter CpG shores were also observed in fluke-positive CCA cases and fluke-negative CCA cases, respectively [2], which highlights the prognostic value of DNA methylation in defining the molecular subtypes of CCA. More recently, genome-wide changes in DNA methylation and enhancer activities have proven valuable in deciphering different molecular subtypes to guide the therapeutic interventions in many other cancers [57,58,59]. In an epigenetic study by Tang and co-workers [60] on intrahepatic CCA, the collagen type XII alpha 1 chain (COL12A1) was identified as a specific biomarker for enrichment in the epithelial–mesenchymal transition pathway and advanced tumor stage. Aberrant expression of COL12A1 was attributed to promoter hypermethylation-induced downregulation of miR-424-5p. The in vivo tumor growth and COL12A1 expression were alleviated by the treatment with the miR-424-5p agonist. This highlighted the potential of epigenetic profiling studies in exploring promising druggable targets for epigenetic therapy of CCA. Taken together, crosstalk between genetic, epigenetic, and PTM is evident in driving the pathogenesis of CCA.

### 3.6. Diagnosis and Evaluation

The clinical manifestations of CCA depend on the location of the tumor within the biliary tract. Extrahepatic tumors present earlier with features of biliary obstruction (jaundice, dark-colored urine, clay-colored stools, and pruritus) in contrast to intrahepatic CCA, where biliary obstruction is less likely. Patients with intrahepatic CCA present late with non-specific symptoms of dull aching abdominal pain, weight loss, or abdominal mass due to a mass effect or invasion into the hepatic parenchyma. Patients presenting with jaundice or upper abdominal pain should undergo liver function tests, which include bilirubin levels (total/conjugated/unconjugated), serum aminotransferases, and alkaline phosphatase (ALP). Patients with extrahepatic CCA will have elevated conjugated bilirubin and alkaline phosphatase. Transaminase levels may also increase in the later course of the disease due to chronic biliary obstruction. Intrahepatic CCAs usually have normal levels of bilirubin but abnormal levels of ALP. The tumor markers include carbohydrate antigen (CA) 19-9 and carcinoembryonic antigen (CEA). However, the sensitivity of these markers is limited for detecting early-stage CCA as these could be elevated in other benign and malignant conditions as well. Alpha-fetoprotein (AFP) can be helpful in differentiating intrahepatic CCA from hepatocellular carcinoma (HCC) as it has high specificity for diagnosing HCC.

Cross-sectional imaging is important to identify the location, extent of involvement, and also to assess the feasibility of surgery. Most patients will undergo transabdominal ultrasonography as obstructive jaundice is a common presentation. Ultrasonography (USG) will help in confirming biliary duct dilation, identifying the site of obstruction and also ruling out gallstones. Multidetector computed tomography (MDCT) is commonly used for diagnosis and staging purposes due to wider availability. Magnetic resonance imaging (MRI) with magnetic resonance cholangiopancreatography (MRCP) provides a non-invasive assessment of the hepatopancreaticobiliary tract. CCA appears as T1 hypointense and T2 hyperintense on an MRI, with proximal ductal dilatation with intense delayed contrast enhancement. MRCP allows for the three-dimensional visualization of bile ducts and vascular structures. It should be performed prior to biliary drainage in order to accurately pick up the pathology. PET or PET/CT does not provide any additional information with respect to the staging of tumors but leads to a change in surgical management due to upstaging in 20–25% of patients [61]. Ductal dilatation of greater than 6 mm with an intact gallbladder in the absence of gallstones points toward a biliary obstructive lesion. Dilatation of the intrahepatic ducts is seen with proximal extrahepatic CCAs, and dilatation of both intrahepatic and extrahepatic ducts may be seen with distal CCAs. An abrupt ductal diameter change suggests tumors at the site of the narrowing. Table 3 describes the radiological findings of CCA with USG, CT, and MRI [62].

Tissue diagnosis can pose challenges, particularly in perihilar lesions. Tissue samples can be obtained by brush cytology, fine needle aspiration, or percutaneous approach. Endoscopic ultrasound (EUS) or endoscopic retrograde cholangiopancreatography (ERCP) is preferred in patients with distal extrahepatic obstruction as it enables the localization and assessment of the extent of the tumor and facilitates brush cytology or biopsy. ERCP has the added advantage of therapeutic intervention, such as stent placement.

### 3.7. Liquid Biopsy in Diagnostics

Liquid biopsies are blood tests for identifying circulating tumor cells, cell-free nucleic acids, and secreted proteins that are found in body fluids such as blood, urine, and bile. Unlike tissue biopsy, liquid biopsy is generally less intrusive, less costly, and safer. There is an increased interest in novel biomarkers from liquid biopsies to guide the diagnosis and treatment of CCA. There have been recent developments in assessing cell-free DNA (cfDNA) as novel CCA biomarkers.

cfDNA is composed of mainly short (50–250 bp) double-stranded DNA fragments that are found in low abundance in body fluids. In healthy individuals, most cfDNA is derived from circulating leukocytes [63]. In cancer patients, a proportion of cfDNA is derived from cancer cells, termed circulating tumor DNA (ctDNA). Depending on the tumor burden, the proportion of cfDNA from a cancer patient’s liquid biopsy can range from less than 1% to as high as 90% [63]. Both real-time quantitative PCR (qPCR) and next-generation sequencing (NGS) platforms can be used to detect mutations in CCA-associated genes (e.g., ARID1A, PBRM1, MTOR, FGFR2, and TP53) in plasma cfDNA from CCA patients [64]. However, the concordance between mutations detected in tumors and those found in cfDNA is highly variable.

cfDNAs from serial liquid biopsies are useful to monitor changes to the tumor mutational profile before, during, and after the treatment period, which can guide designing treatment options. Ettrich et al. [65] analyzed paired plasma cfDNA and tumor samples from CCA patients before and after treatments. They showed that there was a significant concordance between mutations found in tumors and those in the corresponding plasma cfDNA. Furthermore, this study showed that about 63% of the treatment-naive patients had a shift in their mutational profile during treatment, which was also detected in the corresponding ctDNA. Additionally, Goyal et al. [66] showed that cfDNA obtained from serial samplings before and during anti-FGFR therapy contained additional FGFR2 mutations, among other gene mutations. Such cfDNA mutational profiles correlated with those found in the corresponding tumors obtained during the different sampling periods.

Besides plasma, several studies have analyzed bile-derived cfDNA. Driescher et al. [67] showed that bile cfDNA was more sensitive than plasma cfDNA in detecting mutations reported in the matched tumors (96.2% vs. 31.6%). Shen et al. [68] and Arechederra et al. [69] reported significant concordance between mutations detected in tumors and those found in the corresponding bile cfDNA. Apart from detecting CCA-specific mutations in cfDNA, there are currently a few other studies on detecting CCA-specific DNA methylation in cfDNA. Wasenang et al. [70] used a qPCR-based methylation-sensitive high-resolution melting (MS-HRM) approach to assess for the presence of CCA-specific methylated sites on OPCML, HOXA9, and HOXD9 genes in serum-derived cfDNA. Additionally, the Circulating Cell-free Genome Atlas (CCGA) consortium published two studies that used NGS-based targeted methylation panels to study bisulfite-converted cfDNA in a variety of cancers, including CCA [71,72]. They showed that cancer type-specific methylomes were detected in cfDNA albeit with different sensitivity and accuracy for each cancer type. Apart from cfDNA, cell-free RNA, especially microRNA (miRNA) found in exosomes, can be novel CCA biomarker candidates. Recent studies have shown that body fluids, such as plasma and bile, contain a significant amount of miRNAs and some of them could be used to differentiate between CCA patients and healthy individuals [73,74]. Other studies have shown that the expression of miR-21, a known CCA-promoting miRNA, was correlated with tumor stage (late clinical stage) and poorer survival [75]. Furthermore, studies have also identified circulating miRNAs that could potentially be used to differentiate not only CCA from normal samples, but from other types of cancer, especially other hepatobiliary cancers.

In summary, cfDNA and ctDNA from liquid biopsy are attractive diagnostic and monitoring tools for cancer therapy. Despite many positive developments in the use of liquid biopsy for CCA, more research is needed to improve the sensitivity and specificity before its routine clinical usage.

## 4. Current Therapeutic Options

A summary of current and emerging therapeutic options is provided in Table 4.

### 4.1. Surgery

Surgical resection is the primary curative treatment, but it is challenging due to the tumor’s location near the hilar structures. Only about 30% of patients are eligible for successful resection, and even after surgery, there is a high likelihood of disease recurrence, resulting in variable 5-year survival rates of 10–50% depending on the tumor stage and location [76].

A thorough preoperative assessment is vital to determine the feasibility of surgery and to minimize the risk of incomplete resection or non-therapeutic procedures. This assessment should include a review of chronic medical conditions and an evaluation of the future liver remnant (FLR) size and function. Portal vein embolization may be considered for patients with FLR volumes below 20–30% to stimulate hypertrophy of the remaining liver [77]. Key considerations include tumor location, biliary dilation, hepatic lobe atrophy, invasion of hilar vessels, and portal lymphadenopathy [78]. Preparing patients for surgery requires meticulous planning to achieve complete tumor removal (R0 resection). The standard treatment involves removing the affected intra- and extrahepatic bile ducts and the associated hepatic and caudate lobes with the creation of a bilioenteric anastomosis. Two significant factors affecting postoperative complications are preoperative cholangitis and insufficient volume of the FLR, leading to hepatic insufficiency and post-hepatectomy liver failure (PHLF). Preoperative biliary drainage can enhance postoperative liver regeneration, particularly in patients with high bilirubin levels [79]. Although biliary drainage is associated with increased perioperative infection risk, it has been shown to reduce postoperative complications. Achieving bilirubin levels below 2–3 mg/dL is preferable before a major hepatectomy. Ideally, a 6–8 weeks wait between drainage and surgery allows for normalizing impaired hepatic mitochondrial function [80].

Patients with hilar CCA frequently experience nutritional deficiencies that adversely affect their perioperative outcomes. Obstruction of bile flow leads to malabsorption and impaired digestion, particularly of lipids. Additionally, biliary sepsis exacerbates the nutritional compromise. Sarcopenia, characterized by muscle loss, is associated with poor prognosis in liver surgery, and it increases the risk of liver failure in patients undergoing major hepatectomy with extrahepatic bile duct resection for hilar CCA [81]. Identifying the causes of nutritional deficiencies in CCA patients and implementing tailored pre- and postoperative nutritional management is crucial. Surgical resection with negative margins is the best chance of cure and long-term survival. Both radial margin and longitudinal margin should be considered in surgical planning. Occasionally, pancreaticoduodenectomy is also necessary to enable R0 resection. Liver transplantation is considered for selected patients in whom surgical resection is unlikely to achieve negative margins. Exposure can be achieved via various approaches, ranging from the right subcostal incision (often with midline extension), bilateral subcostal incision, or midline incision, depending on surgeon preference and patient body habitus. The dissection begins along the hepatoduodenal ligament to expose the portal structures. The lymph nodes and other extravascular soft tissue may be removed en bloc with the eventual surgical specimen [82]. Next, kocherisation is carried out to assess lymph node metastasis and tumor extension into the pancreatic head. If surgical resection is planned, the distal common bile duct is divided at the pancreas, and a margin is sent for frozen section analysis to determine the need for a pancreaticoduodenectomy (Whipple procedure) to achieve negative margins.

The next step involves assessing the involvement of the portal vein and hepatic artery, which are dissected and evaluated for tumor invasion. The role of preoperative CECT and CE-MRI with MRCP can not be overemphasized in assessing vascular involvement and meticulous preoperative planning of resections. Resectability depends on the extent of involvement of these vessels and their branches. Unresectable CCA refers to CCA tumors that have grown into nearby structures, blood vessels, or organs to the extent that surgical removal is not viable, and it includes most stage III and IV cases. Criteria for unresectability include non-constructible main portal vein involvement, bilateral hepatic artery involvement, unilateral hepatic artery or portal vein involvement with extension into the contralateral biliary tree, or tumor involving the distal common bile duct at the pancreas level, which cannot be managed with concomitant pancreaticoduodenectomy [83,84].

If the tumor is deemed resectable, the surgeon proceeds with hilar resection, often involving hepatectomy (right or left, standard or extended) based on preoperative staging and intraoperative findings. Caudate resection is typically performed to avoid a positive margin of caudate bile ducts [82]. During the procedure, the liver parenchyma is transected using a combination of various techniques, and the ipsilateral hepatic artery and portal vein branches are ligated outside the liver. The liver specimen is carefully demarcated. The intrahepatic bile duct margin assessment usually occurs later in the procedure, and if necessary, additional tissue is excised to ensure negative margins based on frozen section analysis [84].

Following resection, biliary enteric reconstruction is performed using a portion of the jejunum, often requiring a Roux-en-Y reconstruction for a tension-free hepaticojejunostomy. In cases where multiple biliary radicals require reconstruction, adjacent lumens may be joined with sutures to create a single anastomosis. Surgical drains are commonly used, given bile leaks from the liver parenchyma or biliary-enteric reconstruction [83]. Liver transplantation alone is associated with high failure rates in patients with hilar CCA. However, with careful patient selection, excellent outcomes in terms of 5-year recurrence-free survival in the range of 65–70% have been reported in unresectable cases. The criteria for patient selection include positive or strongly suspicious intraluminal brush or biopsy, a radiographic malignant-appearing stricture, elevated levels of CA 19-9, polysomy on FISH (fluorescence in situ hybridization), and a well-defined mass on cross-sectional imaging. A tumor with a radial diameter of more than 3 cm is usually excluded, however, vascular encasement and stricture/mass extension along the duct are not considered contraindications [85,86].

### 4.2. Chemotherapy

The majority of patients with CCA have metastatic or locally advanced disease at presentation. The use of chemotherapy is the standard of care in the management of patients with metastatic and unresectable disease, or recurrent CCA. One of the active regimens for the treatment of advanced biliary tract cancers is cisplatin plus gemcitabine (Gem-Cis), on the basis of the ABC-02 clinical trial [87]. The results of this phase III study demonstrated that the median overall survival was longer for Gem-Cis compared with gemcitabine monotherapy (11.7 months versus 8.1 months; *p* < 0.001). A meta-analysis of two studies using the Gem-Cis based regimen in biliary tract cancer reported that Gem-Cis was associated with a 35% relative reduction in the risk of death risk (HR:0.65, 95% CI: 0.54–0.78, *p* < 0.001) and a 36% reduced risk for disease progression (HR:0.64, 95% CI: 0.53–0.76, *p* < 0.001) and this benefit was present irrespective of the location (CCA vs. gallbladder carcinoma) [88]. A phase III trial has reported that the combination of gemcitabine with S-1 (Gem-S1) is non-inferior to Gem-Cis with a median overall survival (OS) of 15.1 and 13.4 months, respectively (HR = 0.945, 90% CI: 0.78–1.15, non-inferiority *p* = 0.046) [89]. Other agents used in biliary tract cancers are a combination of gemcitabine with oxaliplatin, which has demonstrated clinical efficacy and has a favorable toxicity profile [90], but head-to-head comparisons with Gem-Cis are lacking. Unfortunately, the 5-year survival for CCA patients with metastatic disease is less than 5%. Treatment intensification by using triple combination chemotherapy of gemcitabine, cisplatin, and nab-paclitaxel demonstrated promising response rates and a median OS of 19.2 months at the expense of increased grade 3 toxicity of 58%, mostly neutropenia (33%) [91]. The second triplet regimen explored in this setting is the addition of S1 to Gem-Cis (GCS-1), which led to an improvement in the median OS from 12.6 months to 13.5 months in GCS-1 to Gem-Cis (HR = 0.79, 95% CI: 0.60–1.04, *p* = 0.046). At 1 year, this translated into an absolute survival difference of 5.7% in favor of GCS-1 (59.4% vs. 53.7%) [92]. Table 5 lists the landmark trials with various chemotherapy regimens in the treatment of metastatic/advanced biliary tract cancer.

### 4.3. Adjuvant Chemotherapy

Approximately a quarter to a third of individuals meet the criteria for resection, and following curative-intent surgical resection, there is a 50% 5-year overall survival (OS) rate [95]. However, this positive outcome is counteracted by a considerably high postoperative recurrence rate, ranging between 50 and 70% [96]. Notable adverse prognostic factors include R1 resection and lymph node involvement, prompting the recommendation of postoperative adjuvant therapy for patients with these features [97].

Studies such as BCAT [98] and PRODIGE 12-ACCORD 18 [99] found that both gemcitabine monotherapy and gemcitabine with oxaliplatin therapy did not significantly enhance OS compared to observation. Another pivotal study, BILCAP [100], compared oral adjuvant capecitabine with observation. The per-protocol analysis demonstrated a significant difference in OS between the two groups in favor of capecitabine (median OS: 53 months vs. 36 months, HR = 0.75, 95% CI = 0.58–0.97, *p* = 0.028). A pre-specified intention-to-treat (ITT) analysis, adjusted for nodal status, disease grade, and sex, also revealed a significant difference in favor of capecitabine (HR = 0.71, 95% CI = 0.55–0.92, *p* < 0.01).

In the JCOG1202 ASCOT trial [101], adjuvant S-1 therapy significantly improved OS compared to surgery alone (HR: 0.694, 95% CI: 0.514–0.935, *p* = 0.008). The 3-year OS was 77.1% in the adjuvant arm (95% CI: 70.9–82.1%) and 67.6% for surgery alone (95% CI: 61.0–73.3%). Additionally, the 3-year recurrence-free survival (RFS) was 62.4% for the adjuvant arm (95% CI: 55.6–68.4%) and 50.9% for surgery alone (95% CI: 44.1–57.2%). For a detailed overview of landmark trials in the treatment of resected biliary cancer, please consult Table 6.

### 4.4. Second Line Therapy

Few studies have been conducted on the second-line setting for CCA. In a recent randomized trial, ABC-06 [102], patients with advanced CCA who failed first-line chemotherapy with Gem-Cis were randomized to FOLFOX (oxaliplatin plus 5-FU) and active symptom control. Only patients with a good performance status of ECOG 0–1 were eligible for the study. The use of FOLFOX led to prolongation in the median OS, from 5.3 to 6.2 months (HR: 0.69), and was well tolerated, with increased rates of Grade 3 neutropenia and fatigue. Other agents used in this setting are a combination of capecitabine and irinotecan (XELIRI) [103] and 5FU-based regimens [104].

### 4.5. Radiation Therapy

Radiation plays an important role in all the settings—adjuvant, neoadjuvant, and palliative. The recurrence rates remain high despite surgical resection. The 5-year survival remains poor even after curative resection due to high rates of postoperative recurrence. The most common site of relapse in intrahepatic CCA is the liver (accounting for approximately 60%), with associated extrahepatic relapse in 18.6%, followed by extrahepatic recurrence in 21% [105,106]. The risk factors predictive of recurrence are margin status, lymph node metastasis, lymphovascular invasion, perineural invasion, multiplicity of lesions, and a tumor size greater than 5 cm [107,108].

The pattern of relapse in perihilar CCA includes both local and distant [96,109]. The major prognostic factors are margin status, lymph node metastases, vascular invasion, and tumor differentiation [110,111,112]. In a study by Nakahashi et al. [112], 60% of the patients developed recurrence even after R0 resection. The most common pattern of relapse was locoregional, followed by peritoneum and liver. The probability of recurrence remains high at 50% despite the absence of independent risk factors of recurrence.

Distal CCA has high rates of loco-regional relapse (LRR) despite radical resection. The major factors predictive of recurrence are margin status, lymphovascular invasion, perineural invasion, pancreatic invasion, and lymph node metastasis [113,114].

These statistics indicate that resection alone is not adequate for most of the patients, thus emphasizing the role of adjuvant chemoradiation. Due to the lack of data from phase III randomized trials, the benefit of adjuvant chemoradiation remains unclear. However, various retrospective studies and meta-analyses have supported the role of adjuvant chemoradiation in patients with positive resection margins and lymph node involvement [97,115,116,117,118]. Survival benefit and decreased incidence of LRR was seen in high-risk patients treated with adjuvant chemoradiation [119,120,121]. In a phase II study (SWOG S0809) of adjuvant chemotherapy with gemcitabine and capecitabine followed by chemoradiotherapy with concurrent capecitabine in patients of extrahepatic CCA or gallbladder cancer at high risk of recurrence (pT2-4, node-positive or margin-positive), the combination demonstrated low rates of local failure with acceptable toxicity profile [122]. In a propensity-matched National Cancer Database analysis in patients with distal CCA by Kamarajah et al. [121], adjuvant radiation after surgical resection was associated with a survival benefit regardless of the margin and lymph nodal status. This study emphasized the inclusion of adjuvant radiation in the multimodality treatment of distal CCA. Thus, in patients of extrahepatic CCA with involved margins and lymph nodes and in patients with positive margins in intrahepatic CCA, the guidelines suggest adjuvant chemoradiotherapy in addition to adjuvant chemotherapy [123,124,125,126,127].

The lymphatic drainage area in CCA varies with the location of the tumor [128,129,130]. The target volume should include tumor bed, anastomosis, and regional lymph nodes [128,131]. The lymph node stations to be included in intrahepatic CCA are retroportal, along the hepatoduodenal ligament, common hepatic artery, hilar lymph nodes, retropancreatic, left gastric artery, para-aortic, lesser gastric curvature, and cardial nodes. In perihilar CCA, pericholedochal, hepatoduodenal ligament, retroportal lymph nodes, peripancreatic, common hepatic artery, para-aortic, and those along the left gastric artery are included [128,129]. The lymph node stations to be included in distal CCA are along the common bile duct, common hepatic artery, hepatoduodenal ligament, superior mesenteric artery (SMA), para-aortic, and retropancreatic nodes [129,130].

Radiation can also be used in the neoadjuvant setting to downsize the tumor and convert locally advanced unresectable disease to resectable disease. Based on results by the University of Nebraska [132], the Mayo Clinic [85] developed a neoadjuvant protocol for patients with unresectable, stage I and II perihilar CCA comprising external beam radiotherapy (EBRT) combined with radio-sensitizing chemotherapy with bolus fluorouracil, followed by brachytherapy and maintenance chemotherapy, operative staging to rule out metastases, and liver transplantation. The 5-year survival rate was 82% in patients who underwent liver transplantation. A multicentre retrospective analysis of the experience with neoadjuvant CRT followed by liver transplantation for localized CCA was conducted at 12 large-volume adult liver transplant centers in the United States. Out of 287 patients, 193 were from the Mayo Clinic. Intent-to-treat survival at 2 and 5 years after therapy was 68% and 53%, respectively [133].

In a UCLA study, neoadjuvant therapy was an independent factor affecting prognosis in intrahepatic CCA undergoing orthotopic liver transplantation. Based on the UCLA experience, for patients with a tumor length ≤ 6 cm, conventional radiotherapy with chemotherapy or stereotactic body radiation therapy (SBRT) of 40 Gy in 5 fractions followed by chemotherapy can be considered [134].

Data on the use of SBRT in CCA is emerging. Most of the literature available is from a single institution retrospective series, including patients with intrahepatic and hilar CCA. Various studies have reported high local control rates with acceptable toxicity [135,136]. A meta-analysis of 11 studies evaluating the efficacy of SBRT in unresectable and recurrent CCA demonstrated a one-year local control rate of 78.6% and a one-year OS of 53.8%. The most common side effects were duodenal ulcer and gastric ulcer (incidence of grade ≥ 3 acute toxicity <10 percent) and a late toxicity incidence of 10–20% in most studies [137]. There are no randomized trials comparing SBRT with chemoradiation; however the National Cancer Database evaluated the survival of intrahepatic CCA patients treated with SBRT, chemoradiation, and transarterial radioembolization (TARE). SBRT was associated with a better OS compared to chemoradiation and TARE [138].

Radiation therapy can also be given in palliative settings for alleviating local symptoms and improving the quality of life in patients with locally advanced and metastatic CCA. Different radiation strategies that can be used in palliative settings include conventionally fractionated concurrent chemoradiation, SBRT, hypofractionated radiotherapy, and intraluminal brachytherapy.

The dose of radiation in adjuvant settings is 45 Gy at the rate of 1.8 Gy per fraction and boosts the tumor bed to 54 Gy to 59.4 Gy, depending on margin status. Most centers prefer giving capecitabine in concurrent settings. In the neoadjuvant settings, a dose of 45 to 50.4 Gy is delivered with capecitabine or gemcitabine-based chemotherapy. SBRT is given in the range of 30 to 50 Gy in 3 to 5 fractions depending on the disease status and ability to achieve normal tissue constraints.

## 5. Emerging Therapeutic Strategies

A summary of the studies focusing on emerging therapeutic options and their targets are summarized in Table 7.

### 5.1. Targeted Therapies

Traditional chemotherapy has limited effectiveness in treating CCA; therefore, targeted therapeutic approaches for CCA from different etiologies are of high clinical need to improve patient outcomes. So far, the FDA has approved three types of drugs for the treatment of advanced CCA. The first is for the inhibition of FGFR1-3, and the drugs approved are pemigatinib (Pemazyre, Incyte Corporation, USA), infigratinib (Truseltiq, QED Therapeutics, USA), and futibatinib (Lytgobi, Taiho Pharmaceutical, Japan). FGFR2 fusions and rearrangements are more prevalent in fluke-negative CCA and are predominantly seen in intrahepatic CCA [56,139]. Clinical trials have shown that with the usage of pemigatinib for CCA patients with FGFR2 rearrangement, the overall objective response is 35.5% [140]. Other clinical trials have shown that infigratinib is effective for pretreated patients and in the first-line setting [141]. The second approved drug, ivosidenib (Tibsovo, Servier Pharmaceuticals, USA), is a selective inhibitor of IDH1. IDH1 mutations are also more commonly found in fluke-negative CCA [2,142]. In clinical trials, ivosidenib has been demonstrated to improve progression-free survival in patients with previously treated IDH1-mutant CCA [143]. The use of immune checkpoint inhibitors has also emerged as a promising targeted therapeutic approach, and recently, the FDA approved the use of durvalumab in combination with gemcitabine and cisplatin for patients with advanced or metastatic CCA. Clinical trials have shown that durvalumab in combination with chemotherapy significantly improves OS and PFS for these patients [144].

Other than these FDA approved drugs, there are other promising targeted therapies still under development for the treatment of fluke-negative CCA. For example, another IDH inhibitor, enasidenib, is in clinical trials for CCA patients [145]. Preclinical studies have shown that EGFR inhibitors, such as erlotinib, can inhibit the growth of CCA cells in vitro [146]. Tazemetostat, an inhibitor of EZH2, commonly found overexpressed in CCA, has shown effectiveness in CCA models in vitro [147]. Bintrafusp alfa is a first-in-class bifunctional fusion protein that targets both PD-L1 and TGF-β. Currently, a phase I study of bintrafusp alfa in patients with pretreated CCA has promising results and further studies are being planned [148]. Nicotinamide N-methyltransferase (NNMT) has been identified as an oncogene. As the master regulator of NAD homeostasis, it will potentially affect all NAD-dependent enzyme activity [149]. NNMT has been found to be overexpressed in CCA, which contributes to tumor progression and metastasis [150], thus representing a promising molecular target. Many NNMT inhibitors have been developed which could be used for this aim.

In contrast, there are currently no FDA-approved drugs for the treatment of fluke-positive CCA. HER2/ERRB2 amplifications are more common in fluke-positive CCA [2]. Zanidatamab, a bispecific antibody for HER2, has received FDA breakthrough therapy designation for patients with locally advanced, unresectable, or metastatic HER2-expressing CCA. In a Phase 2b HERIZON-BTC-01 trial, zanidatamab has been reported to show an objective response rate of 41.3% in patients with previously treated HER2 amplified and expressing CCA [151]. With a dearth of effective targeted therapies for fluke-positive CCA, the standard of care for these patients remains resection and chemotherapy [152].

### 5.2. Immunotherapy

Several preclinical studies have studied the immune system and the tumor microenvironment, including the stromal cells and their surrounding secretory cytokines, to understand the usefulness of immunotherapy in CCA. Tumor cells express the ligand PD-L1 to escape an attack from the immune T cells through the PD-L1/PD-1 axis by promoting apoptosis [153]. Increased expression of PD-L1 is associated with the pTNM stage and inferior overall survival and is inversely correlated with CD8+ TILs in CCA [154,155]. CD3+ and CD8+ T lymphocyte infiltration have resulted in superior survival with reduced local recurrences and Tregs infiltration co-related with inferior survival in patients with CCA who underwent resection [156].

There has been growing evidence of increased PD-1 and CTLA-4 expression on the lymphocytes of T cells in CCA, which could be potentially targeted by immune checkpoint inhibitors. Next-generation sequencing and single-cell RNA sequencing (scRNA seq) of CCA have paved the way to identify novel immune subsets and pathways that can be explored for potential targets. A study using scRNA seq showed an increase in inhibitory checkpoint receptors like T cell immunoglobulin and mucin-containing protein 3 (TIM3), lymphocyte-activation gene 3 protein (LAG-3), and T cell immunoreceptor with Ig and ITIM domains (TIGIT) on the CD8 T cells in CCA [157]. Similarly, Tregs in CCA have been shown to express these inhibitor markers, like TIGIT, CTLA-4, and the TNFR-related protein superfamily [157], making them more susceptible to the antitumor effect of PD1 blockade.

Despite evidence suggestive of a microenvironment conducive to favorable immunotherapeutic outcomes in CCA, the results have been disappointing. For over a decade, there has been no phase III data that has shown an improvement in survival outcomes after the ABC-02 in 2010, until the recent TOPAZ-1 trial data, with an improvement in survival by a few weeks [144].

In patients with CCA, around 2–3% had microsatellite instability (MSI-H) tumors, and 4–6% with a high tumor mutation burden (TMB-H) were noted [158,159]. TMB-H and MSI-H tumors are a distinct subset, with increased responses to immune checkpoint blockade due to increased infiltration of immune cells and release of tumor-specific neo-antigens. A phase II study (NCT01876511) evaluating anti-PD1 immunotherapy in advanced carcinoma, including ampullary and CCA patients, showed promising responses in MSI-H CCA [160]; based on these, the study was expanded to include more patients with advanced mismatch repair deficiency of 12 different cancer types including CCA, and noted favorable responses as the tumor-induced neoantigens in MSI-H group improved immunotherapeutic outcomes in CCA similar to other cancer types [161]. These results led to the approval of anti-PD1 immunotherapy in adult solid tumors with MSI-H, including CCA, after progression on first-line therapy.

Pembrolizumab monotherapy in the KEYNOTE-158 study showed activity in metastatic MSI-H solid tumors, including CCA. There were 22 CCA patients in the study who had progressed on prior treatment and were found to have MSI-H. The objective response rate (ORR) in the CCA cohort was 40.9%, with two complete responses and seven partial responses. The median PFS and OS were 4.2 months and 24.3 months, respectively, similar to that reported in other cancer subsites. Monotherapy with nivolumab has shown activity in MSI-H CCA [162]. In the majority of CCA patients who are MSS or low TMB, there has been a wide array of studies to explore an ideal biomarker for response to immune checkpoint inhibitors. PD-L1 level of expression was used, like in other cancers, as a marker of response to immunotherapy. The KEYNOTE-028 showed disappointing results with pembrolizumab monotherapy in CCA patients with PD-L1 IHC > 1% positive tumors. Similarly, the KEYNOTE-158, including patients with CCA, showed a suboptimal ORR of 6.6% and 2.9% in the PDL1 + and PDLI negative patients [163]. Nivolumab, a PDL-1 inhibitor, has shown modest improvement in PFS in the PDL-1+ subset in a phase II trial [162]. Despite a positive correlation between the level of expression and response rate, both nivolumab and pembrolizumab have modest clinical benefits in CCA and there is a need to better understand their resistance mechanisms.

Unlike other cancer subtypes like non-small cell lung cancer (NSCLC), bladder, or renal cell cancer, combinatorial approaches of chemoimmunotherapy or adding VEGF inhibitors or local ablation to immunotherapy can result in improved outcomes for patients with CCA. This rationale led to the early phase II study, which combined nivolumab with gemcitabine and cisplatin chemotherapy, which was the standard of care and resulted in an ORR of 61.9%, with 18.6% of patients achieving a CR. The median PFS was 6.1 months, and the mOS was 8.5 months, with a manageable toxicity profile [164].

The TOPAZ-1 trial [144], which combined an anti-PDL1 antibody, durvalumab with gemcitabine and cisplatin chemotherapy followed by durvalumab maintenance, resulted in a statistically significant improvement in OS with a median OS of 12.8 months versus 11.5 months in the placebo treatment group. The toxicity profile was manageable, with no significant difference in survival outcomes based on the level of PDL-1 expression in the post hoc analysis. This promising data led to the approval of a new standard of care in the first-line treatment of advanced biliary tract and CCA.

Furthermore, studies have explored the combination of anti-PD1/PDL-1 with anti-CTLA 4 to achieve deeper prolonged responses in a phase II trial that combined ipilimumab with nivolumab in patients with recurrent biliary tract MSS tumors and documented median PFS of 2.9 months and OS of 5.7 months [165].

Camrelizumab, an anti-PD1 antibody plus gemcitabine and oxaliplatin combination chemotherapy in first-line treatment of advanced biliary cancers, resulted in a median PFS and median OS of 6.1 months and 11.8 months, respectively [166]. Another chemoimmunotherapy combination of camrelizumab with FOLFOX or GEMOX showed an ORR of 16.6%, and the median PFS and OS were 5.3 months and 12.4 months, respectively [167]. There have been various other drugs like VEGF inhibitors (axitinib, lenvatinib, ramucirumab, and regorafenib) and PARP inhibitors (olaparib, rucaparib) in early-phase clinical trials being tested in combination with immunotherapy to improve outcomes in patients with advanced cholangiocarcinoma.

**Table 7:** Summary of emerging therapeutic options and their targets.

Type of Therapy	Drug	Publication	Target
Targeted therapies	Enasidenib	Reference [145]	IDH2
	Tazemetostat	Reference [147]	EZH2
	Bintrafusp alfa	Reference [148]	TGF-β and PD-L1
	Nicotinamide N-methyltransferase inhibitors	Reference [150]	NNMT
Immunotherapies	Pembrolizumab	Reference [163]	PD-1
	Nivolumab	Reference [162]	PD-1
	Anti-PD1/PD-L1 + anti-CTLA4	Reference [165]	PD-1/PD-L1, CTLA4
	Camrelizumab	Reference [167]	PD-1
Combination	Camrelizumab + gemcitabine + oxaliplatin	Reference [166]	PD-1, DNA synthesis, DNA damage

Although the data from the TOPAZ-1 study showed an improvement in overall survival with the addition of immunotherapy to chemotherapy, it resulted in a prolongation of survival by only a few weeks. The presence of the dense desmoplastic stroma and fibroblast-rich tumor micro-environment in CCA has resulted in resistance to many chemotherapeutic drugs, including immune checkpoint inhibitors. The lack of clinical trials due to the low incidence of CCAs, geographical variability, and multiple sub-sites have made patient recruitment challenging and hamper access to newer therapies.

There have been significant differences in chemo responsiveness, histology, and mutational profile between the intrahepatic, perihilar, and distal CCA, which has made detection of ideal biomarkers challenging. Identification of predictive and prognostic biomarkers will help choose the right patients who seem to derive the maximum benefit. Incorporation of the high throughput sequencing data and omics, including proteomics and metabolomics, will help in better understanding the biology of the disease and provide enormous information on the drug-resistant mechanisms in CCAs.

Despite these limitations, there have been various clinical trials in the pipeline harnessing the role of second-generation immunotherapeutic drugs like anti-TIM3, anti-TIGIT, and TGF beta in combination with chemotherapy and anti-PD1 in CCA.

### 5.3. Novel Compounds from Natural Sources

Over the last two decades, considerable research has been carried out to find drugs from natural sources, e.g., medicinal plants, marine, fungi, isolated compounds, and their semi-synthetic derivatives, for the treatment of CCA. Researchers have applied different approaches, such as repurposing of plant extracts/compounds, herbal formulations, reverse pharmacology, synergistic interaction between compounds and anticancer drugs (5-FU and gemcitabine), isolation of novel molecules, and preparation of semi-synthetic analogues in search of a therapeutically potent candidate against CCA. Several diverse natural scaffolds with promising anti-CCA activity were subsequently isolated. Significantly, an oral formulation from the standardized extract of Atractylodes lancea rhizomes has completed a Phase I clinical trial with a satisfactory safety profile, and a Phase II clinical trial in patients with advanced-stage CCA is ongoing [168].

About 93 compounds with anti-CCA activity (79 compounds were isolated from plants and 19 compounds from fungi) are shown in Table 8. Chemical classes, including flavonoids (pongaflavone, candidone, 3,7,3′,4′-tetramethoxy flavones), chalcones (tunicatachalcone, obovatachalcone), naphthoquinone ester (rhinacanthin-C), caged xanthones (isomorellin, isomorellinol, forbesione, gambogic acid), bisbenzylisoquinoline alkaloid (tiliacorinine), sesquiterpenes (phomoarcherin B, phomoarcherin C), and depsidones (mollicelline K, mollicelline N, mollicelline C, mollicelline F) emerged as promising hits with half-maximal inhibitory concentration (IC_50_) < 10 μg/mL against CCA cell-lines [169,170,171,172,173]. However, only four compounds have been investigated in animal models (see Table 8). Further research, including in vivo pharmacological studies along with the mechanism of action is encouraged for validating the safety and efficiency of the natural-derived compounds in the treatment of cholangiocarcinoma.

### 5.4. Modulating Gut Microbiota

#### 5.4.1. Microbiota Dysbiosis in CCA

Recent research has shown that the progression of precancerous diseases to CCA is significantly correlated with microbiota dysbiosis. Numerous research studies have revealed that the microbiota are potentially useful as biomarkers for CCA. Deng et al. [177] investigated the gut microbiota differences between patients with CCA, hepatocellular carcinoma (HCC), and healthy controls. They discovered that 11 gut microbiome species were significantly enriched in CCA patients (Table 9). Zhang et al. [178], reported that Burkholderia, Cabelleronia, Paraburkholderia, Faecalibacterium, and Ruminococcus-1 genera in gut microbiota are significantly enriched in the gut microbiota of CCA patients. Lactobacillus, Actinomyces, Peptostreptococcaceae, Alloscardovia, and Bifidobacteriaceae families are found abundant in patients with intrahepatic CCA (iCCA) and can be employed as biomarkers to distinguish iCCA patients from healthy controls [179]. Notably, Faecalibacterium, Rumminococcus, Lactobacillus, Burkholderia, Caballeronia, and Paraburkholderia genus were the most common bacteria species in CCA patients. Additionally, they discovered that six conjugated bile acids (BAs) were significantly increased in the plasma of iCCA patients, whereas chenodeoxycholic acid in plasma was significantly decreased.

With high-throughput sequencing technology, current research studies have discovered the alterations in the bile and oral microbiome that may be linked to the development of CCA. Bile samples from different types of CCA were collected and compared for the bile microbiome of CCA. The study found that Pseudomonas, Sphingomonas, and Halomonas genera were the most dominant in the bile of patients with pCCA, whereas Streptococcus, Prevotella, and Halomonas genera were the most abundant in dCCA patients and Pseudomonas, Chloroplast, and Acinetobacter were the most dominant in pancreatic cancer [180]. The oral microbiota of CCA and HCC patients was studied using 16S rRNA sequencing. Lautropia, Alloprevotella, and Actinomyces families were shown to be particularly common microbiomes in CCA patients compared to healthy controls [181]. Even though microbiota can be used to distinguish CCA patients and those with different diseases and, therefore, can be used as markers for non-invasive CCA diagnostic tools, the connection between the microbiota and CCA is poorly understood, and more studies are needed to understand alterations and mechanisms.

#### 5.4.2. Modulating Gut Microbiota in CCA Patients

There is increasing evidence that dysbiosis of the gut microbiome could promote tumorigenesis and influence CCA development. Gut microbiota modulation aimed at restoring gut microbial homeostasis has emerged as a possible strategy for CCA prevention and treatment responses. We summarize various gut microbiota modulation strategies, such as probiotics, prebiotics, and fecal microbiota transplantation (FMT) in CCA prevention.

#### 5.4.3. Probiotics in CCA Prevention

Probiotics, which are defined as living microorganisms that confer health benefits on the host, have been shown in recent studies to restore microbial dysbiosis and maintain intestinal microbial balance by occupying host tissue and preventing pathogenic bacteria colonization. Currently, three randomized-controlled trials are ongoing to understand the effect of probiotics in hepatobiliary tumors, including CCA and HCC (Table 10). The results showed that TNFα and interleukin 1β levels significantly increased in patients who were orally administered the mixed probiotics (containing Bifidobacterium lactis, Lactobacillus plantanum, and Lactobacillus salivarius) in the post-operative periods, thereby supporting the use of probiotics to modulate the immune response in reducing complications of infections in patients [182]. However, there is no current evidence to support the mechanism of probiotics modulating gut microbiota in CCA patients, and, therefore, more clinical studies are needed to prove the therapeutic benefits of probiotics in CCA patients.

#### 5.4.4. Prebiotics in CCA

Prebiotics are non-digestible food substances that specifically increase the growth and/or activity of particular bacteria in the gut and improve human health. Non-digestible food ingredients include typical carbohydrate and fiber-based prebiotics, polyunsaturated fatty acids (PUFAs), and polyphenols [183]. Several studies in recent years have found that prebiotics increase the abundance of specific probiotics in the gut, particularly beneficial bacteria, such as Lactobacillus, Bifidobacterium, Faecalibacterium, Akkermansia, Ruminococcus, and Rosebura species [184,185,186,187]. The enrichment of beneficial probiotics in the gut is linked to pathogen protection and immune response modulation. However, research on the direct effects of prebiotics on CCA is scarce. A recent clinical trial has investigated the effect of PUFA eicosapentaenoic acid supplementation on gut microbiota modulation to increase anti-cancer immune response in patients with colorectal cancer liver metastasis (Table 10).

#### 5.4.5. Fecal Microbiota Transplantation (FMT) in CCA

Another emerging treatment method for gut microbiota modulation is FMT. FMT is the transfer of the complete fecal microbial community, including bacteria, viruses, fungi, and their metabolites, from a healthy donor to a recipient and has been recognized as a potential therapeutic strategy for restoring microbial balance in a variety of gastrointestinal illnesses, including irritable bowel syndrome, IBD, and Clostridium difficile infection [188]. However, there are no clinical trials evaluating the benefits of FMT on gut microbiota modulation for the prevention and treatment of CCA. A recent clinical trial is currently recruiting patients to study the safety and efficacy of FMT in combination with atezolizumab and bevacizumab in HCC patients who have not responded to immunotherapy (Table 10). Twelve patients have been recruited in total so far. The effect of FMT on recipient gut microbiota composition, immunological activity, and serum and stool metabolomic and lipidomic markers before and after FMT will be investigated.

#### 5.4.6. Implications of Recent Research for the Development of Future CCA Therapeutics

Recent advances in CCA research hold optimistic prospects for the future development of therapeutics. Notable advancements in clinical trials showcasing the effectiveness of targeted and immunotherapy for CCA have the potential to refine treatment precision and improve outcomes. The synergy of combination therapies may enhance efficacy while minimizing adverse effects. The emergence of alternative therapies, such as using novel compounds or modulating gut microbiota, offers a diversity of effective treatment options for patients. Importantly, recent progress may improve patient outcomes, leading to extended survival rates and an enhanced quality of life. Despite these breakthroughs, challenges in translating laboratory findings into successful clinical trials emphasize the necessity for ongoing research. Nevertheless, ongoing research holds considerable potential for advancing the field and optimizing treatment strategies for CCA.

## 6. Conclusions

Managing hilar cholangiocarcinoma requires the expertise of multiple medical disciplines, including hepatobiliary surgery, transplant surgery, medical oncology, radiation oncology, diagnostic radiology, interventional radiology, gastroenterology, and pathology. Standardizing best practices and advancing translational research efforts are crucial for expanding treatment options and tailoring choices for patients with hilar cholangiocarcinoma.

The molecular pathobiology of CCA has been extensively studied in depth in recent years, leading to the development of therapies that have shown promise for treatment and the emergence of FDA-approved drugs. Despite the significant advances in the field of CCA, there is still much work to identify other novel targets and optimize the efficacies of existing therapies. With continued research and clinical efforts, the goal is to improve the prognosis and quality of life for patients with CCA. It is essential for early drug developmental researchers, clinical scientists, bioinformatics statisticians, and clinical oncologists to work in close proximity to identify actionable biomarkers, pathways of drug resistance, and to decipher druggable targets to improve clinical outcomes in patients diagnosed with advanced CCA.

## Figures and Tables

**Table 1:** Summary of CCA risk factors from East Asian and Western Countries.

East Asian Countries	Western Countries
*Opisthorchis viverrini (Ov)*	Primary sclerosing cholangitis (PSC)
*Clonorchis sinensis*	Choledochal cysts
	Caroli disease
	Caroli syndrome
	Liver cirrhosis
	Cholelithiasis
	Choledocholithiasis
	Hepatitis B virus (HBV)
	Hepatitis C virus (HCV)
	Non-alcoholic fatty liver disease (NAFLD)
	Type 2 diabetes mellitus
	Inflammatory bowel disease
	Alcohol consumption
	Tobacco smoking
	Obesity
	Hypertension

**Table 2:** Prevalence of genetic aberrations in intrahepatic and extrahepatic cholangiocarcinoma (adapted from Refs. [2,54].

Genetic Aberrations	Intrahepatic CCA (%)	Extrahepatic CCA (%)	Normal Function of Gene	Mechanisms
**Inactivating mutations**				
*TP53*	20–40	30–60	Tumor suppressor	Transcription factor.
*ARID1A*	7–36	12–20	Tumor suppressor	Transcription factor.
*CDKN2A/B*	6–27	9–28	Tumor suppressor	Cell cycle.
*BAP1*	13–21	1	Tumor suppressor	Transcription factor.
*SMAD4*	4–17	10–22	Tumor suppressor	Transcription factor
*RNF43*	9	1	Tumor suppressor	Wnt signaling.
*PTEN*	1–11	1	Tumor suppressor	Membrane binding.
**Activating mutations**				
*KRAS*	7–24	8–45	Proto-oncogene	Signaling cascade, e.g., MAPK pathway.
*IDH1/2*	10–30	0–7	Proto-oncogene	Glucose metabolism, cellular defense against oxidative stress.
*PIK3CA*	3–9	5–7	Proto-oncogene	Cell growth, survival, and motility.
*BRAF*	3–7	3–7	Proto-oncogene	Signaling cascade, e.g., RAS/MAPK pathway.
**Amplifications**				
*ERBB2/3*	4–8	5–17	Proto-oncogene	Signaling cascade, e.g., PI3K/AKT and ERK signaling.
*MET*	2–7	1	Proto-oncogene	Tyrosine kinase.
**Fusions**				
*FGFR1-3*	10–45	1	Proto-oncogene	Cell proliferation, differentiation, migration, and apoptosis
*NTRK*	4	4	Proto-oncogene	Receptor tyrosine kinases.

**Table 3:** Radiological findings of CCA with USG, CT, and MRI.

Location	USG	CT	MRI
Intrahepatic	Mass with irregular margins, which may be hypo- or hyperechoic or may have mixed echogenicity.	Hypodense lesion in the liver, which can be well-defined or infiltrative, with bile duct dilatation. Capsular retraction may be seen in 20% of cases. Peripheral rim enhancement in both the arterial and venous phases.	T1 hypointense and T2 hyperintense mass with proximal ductal dilatation with intense delayed contrast enhancement.
Perihilar	Ductal dilatation of both intrahepatic ducts or nonunion of the right and left hepatic ducts	Ductal dilatation of both intrahepatic ducts or nonunion of the right and left hepatic ducts.	Presence of mass on the central hepatic ducts, prominent intrahepatic duct dilatation. Best visualized on delayed imaging (obtained 1–5 min after contrast administration).
Distal	Indirect signs: ductal dilatation	Dilatation of intrahepatic and extrahepatic ducts with distended gall bladder. Abrupt change in ductal diameter.	Circumferential thickening and delayed enhancement of the bile duct wall. Abrupt change in the ductal diameter. Best visualized on delayed imaging (obtained 1–5 min after contrast administration).

**Table 4:** Summary of current and emerging therapeutic options.

Current	Emerging
**Surgical Resection**	**Targeted therapies** - Enasidenib - Tazemetostat - Bintrafusp alfa - Nicotinamide N-methyltransferase inhibitors
**Chemotherapy** - Cisplatin + gemcitabine - Gemcitabine + S-1 - Cisplatin + gemcitabine + S1 - Gemcitabine, cisplatin, and nab-paclitaxel	**Immunotherapy** - Pembrolizumab - Durvalumab - Anti-PD1/PD-L1 + anti-CTLA4 - Camrelizumab
**Adjuvant Chemotherapy** - Capecitabine - S-1	**Combination** - Camrelizumab + gemcitabine + oxaliplatin - Immunotherpay + VEGF inhibitors - Immunotherapy + PARP inhibitors
**Second line therapy** - Oxaliplatin + 5-FU - 5FU - Capecitabine + irinotecan	**Novel compounds**
**Radiation therapy** (including neoadjuvant)	**Gut microbiota**
**Targeted therapies** - Pemigatinib (Pemazyre) - Infigratinib (Truseltiq) - Futibatinib (Lytgobi) - Ivosidenib (Tibsovo)	
**Combination** - Durvalumab + gemcitabine + cisplatin	

**Table 5:** Randomized phase III clinical trials evaluating adjuvant chemotherapy in advanced/metastatic biliary tract cancer.

Trial	Arm	No. of Patients	Biliary Cancer Subtype	Outcome Median OS, HR
ABC-02 [87]	Gemcitabine 1000 mg/m^2^, cisplatin 25 mg/m^2^ on days 1 and 8, every 3 weeks × 8 cycles vs. gemcitabine 1000 mg/m^2^ days 1, 8, and 15, every 4 weeks × 6 cycles.	410	58% Cholagiocarcinoma	11.7 months vs. 8.1 months 0.640 (0.52–0.80), *p* < 0.001
FUGA-BT (JCOG1113) [89]	Gemcitabine 1000 mg/m^2^, cisplatin 25 mg/m^2^ on days 1 and 8, every 3 weeks vs. gemcitabine 1000 mg/m^2^ infusion on days 1 and 8 and S-1 orally twice daily 60–80 mg/day on days 1–14, every 3 weeks till progression.	354	57% Cholangiocarcinoma	13.4 months vs. 15.1 months 0.945 (0.78–1.15) Non-inferior
Kim et al. [93]	Gemcitabine 1000 mg/m^2^ on days 1 and 8, and oxaliplatin 100 mg/m^2^ on day 1, every 3 weeks × 8 cycles vs. capecitabine 1000 mg/m^2^, twice daily, on days 1–14 and oxaliplatin 130 mg/m^2^ on day 1, every 3 weeks × 8 cycles.	222	74% Cholangiocarcinoma	10.4 months vs. 10.6 months *p* = 0.131
KHBO1401-MITSUBA [92]	Gem Cis vs. Gem Cis S1 gemcitabine 1000 mg/m^2^ and cisplatin 25 mg/m^2^ infusion on day 1. gemcitabine 1000 mg/m^2^ and cisplatin 25 mg/m^2^ infusion on day 1, and 80 mg/m^2^ of S-1 on days 1–7, every 2 weeks.	246	64–65% Cholangiocarcinoma	13.5 months vs. 12.6 months 0.79 (0.628–0.996), *p* = 0.046
SWOG 1815 [94]	Gemcitabine 800 mg/m^2^, cisplatin 25 mg/m^2^, Nab paclitaxel 100 mg/m^2^ on days 1 and 8, every 3 weeks vs. gemcitabine 1000 mg/m^2^, cisplatin 25 mg/m^2^ on days 1 and 8, every 3 weeks till progression.	441	84% Cholangiocarcinoma	14.0 months vs. 12.7 months 0.93, (0.74–1.19), *p* = 0.58

**Table 6:** Randomized phase III clinical trials evaluating adjuvant chemotherapy in resected biliary tract cancer.

Trial	Experimental	Control	No of Patients	Biliary Cancer Subtype	Outcome
BCAT [98]	Gemcitabine 1000 mg/m^2^ D1, 8, and 15, every 4 weeks × 6 cycles.	Observation	117 108	All extrahepatic cholangiocarcinoma	Primary outcome: OS not significant (*p* = 0.964), median survival 62.3 vs. 63.8 months, HR = 1.01.
PRODIGE12/ACCORD18 [99]	Gemcitabine 1000 mg/m^2^ day 1 + oxaliplatin 85 mg/m^2^ day 2, every 2 weeks × 12 cycles.	Observation	73 82	80% Intra and extrahepatic cholangiocarcinoma	Primary outcome: RFS not significant (*p* = 0.48), median relapse-free survival 30.4 vs. 18.5 months, HR = 0.88.
BILCAP [100]	Capecitabine 1250 mg/m^2^ day 1–14, every 3 weeks × 8 cycles.	Observation	210 220	82% Intra and extrahepatic cholangiocarcinoma	Primary Outcome: OS Significant by per-protocol analysis (*p* = 0.028), median survival 53 vs. 36 months, HR = 0.75.
JCOG 1202 [101]	S-1 40 mg, 50 mg, or 60 mg according to body surface area, orally administered twice daily for 4 weeks, followed by 2 weeks of rest for four cycles.	Observation	218 222	69% Intra and extrahepatic cholangiocarcinoma	Primary outcome: OS significant (*p* = 0.0080), median overall survival was not estimable vs. 6.1 years, HR: 0.69.

OS: overall survival; RFS: relapse-free survival; HR: hazard ratio.

**Table 8:** Natural compounds studied for anti-cholangiocarcinoma effect in animal models.

Compounds (Class)	Source	Doses	Pharmacological Effects	Mechanism of Action	Reference
Tiliacorinine (bisbenzylisoquinoline alkaloid)	*Tiliacoratriandra*(Colebr.) Diels (roots and stems)	CCA-xenografted mice dosage: 10 mg/kg body weight, once daily for 3 consecutive days. Route: intraperitoneal injection.	Reduced tumor volumes 45.16 ± 12.52 mm^3^ and tumor weight 0.07 ± 0.02 g compared to the control group (injected with 0.01% DMSO) 80.22 ± 18.75 mm^3^ and 0.13 ± 0.04 g, respectively.	apoptosis induction via cleavage of PARP-1 by caspase, BAX -Bcl_xL_ and XIAP	Janeklang et al. [170]
Thymoquinone (1,4-benzoquinones)	*Nigella sativa* L. oil	Xenografted nude mice. dosage: 2, 4, or 8 mg/mouse for 20 days. Route: intragastric intubation	Reduced tumor size compared to control (PBS) groups.	PI3K/Akt and NF-κB and regulated gene products, including *p*-AKT, p65, XIAP, Bcl-2, COX-2, and VEGF.	Xu et al. [174]
Streptochlorin (indole alkaloid)	Marine *Streptomyces* sp.	HuCCT-1-xenografted nude mouse. Dosage: 5 mg/kg for 22 days. Route: subcutaneously injection.	Inhibited tumor growth (tumor volume 6.6 times and 5.4 times smaller than control (phosphate-buffered saline) and vehicle (thermosensitive gel) groups). Inhibited invasion and migration of CCA cells. Regulated tumor metastasis of HuCCT-1 cells in mouse liver metastasis.	Apoptosis induction -Bcl-2 expression Bax, Bad, and cytochrome c activation of caspase-3. -NFkB, VEGF, and Notch 1	Kwak et al. [175]
Epigallocatechin gallate (EGCG) (catechin)	Green tea	Mz-ChA-1 cells xenografted nude mice. Dosage: 20 mg/kg EGCG for 10 days and a combination study of i.p. injections of EGCG (20 mg/kg) for 10 days, and gemcitabine (120 mg/kg) for 3 h following EGCG injections on days 1, 4, and 7 only. Route: intraperitoneal injections.	Reduced the growth of tumor and increased the sensitivity to Gemcitabineof Mz-ChA-1 cell xenografts in nude mice EGCG alone showed a significant reduction in tumor growth compared to gemcitabine.	Induction of apoptosis.	Lang et al. [176]

**Table 9:** Dysbiosis of the microbiota of cholangiocarcinoma and its precancerous diseases.

Hepatobiliary Diseases	Bacteria	Location	Metabolites	Associated Cancer Mechanism	Reference
CCA patients (n = 46), hepatocellular carcinoma (HCC) patients (n = 143).	Enriched in ***Faecalibacterium***, *Klebsiella*, ***Ruminococcus*** *Gnavus* group, ***Lactobacillus***, *Dorea*, *Veillonella*, ***Burkholderia***, ***Caballeronia***, ***Paraburkholderia***, and *Citrobacter* genera.	gut	NA	The microbial biomarker of CCA	Deng et al. [177]
pCCA (n = 14), dCCA (n = 9), Pancreatic cancer (PC) (n = 8). and cholelithiasis (n = 22).	The top three biomarkers for pCCA in the genus level were *Pseudomonas, Sphingomonas,* and *Halomonas*; for dCCA, were *Streptococcus, Prevotella, and Halomonas*; and for PC were *Pseudomonas, Chloroplast,* and *Acinetobacter.*	bile	Differences in the metabolic pathways in different groups when compared to healthy controls.	The correlation between bile microbiome and the progression of pCCA, dCCA, and PC.	Li et al. [180]
CCA (n = 74), HCC (n = 35).	The diagnostic biomarkers for CCA were *Lautropia, Alloprevotella*, and *Actinomyces.*	oral microbiome	NA	The oral microbial markers for non-invasive diagnostic tools for CCA	Rao et al. [181]
CCA patients (n = 53), cholelithiasis (n = 47).	Enriched in ***Burkholderia, Cabelleronia, Paraburkholderia, Faecalibacterium***, and ***Ruminococcus-1*** genera.	gut	NA	The gut microbiota served as a non-invasive diagnostic biomarker for early diagnosis of CCA.	Zhang et al. [178]
iCCA patients.	Enriched in ***Lactobacillus***, * Actinomyces, Peptostreptococcaceae, Alloscardovia*, and *Bifidobacteriaceae* family.	gut	Increased in six conjugated bile acids (BAs) and decreased in chenodeoxycholic acid in plasma of iCCA patients.	Altered gut microbiota correlated with the BAs metabolism and inflammatory cytokines.	Jia et al. [179]

**Table 10:** Clinical trials of modulation of gut microbiota in hepatobiliary tumors (CCA, HCC, and gallbladder cancer).

Condition/Disease	Sample Size (n)	Intervention	Outcomes	Status (Location)	NCT Number	References
1. **Probiotics**						
Liver cancer	46	The oral probiotic (*Lactobacillus rhamnosus*) administered one time a day during the immunotherapy treatment within 6 months.	NA	Recruiting (Jiangxi Provincial Cancer Hospital)	NCT05032014	-
HCC in cirrhosis	280	Orally administered 5 mL of probiotics (containing *L.casei, L. palntarum, Strptococcus faecalis,* and *Bifidobacterium*) every 12 h for 10 consecutive days per month. Duration of treatment: 2 cycles of continuous treatment per year during a 3-year period (6 cycles).	NA	Not yet recruiting: Austral University, Argentina	NCT03853928	-
Liver fibrosis Liver cirrhosis HCC	664	Administered active substance mixture of *Bifidobacterium lactis* LA303, *L.plantanum* 301, * L.salivarius* LA, and *Bifidobacterium lactis* 304. 2 capsules per day for 14 days.	The administration of probiotics promoted an immune response in patients in the post-operative period.	Completed: University Hospital Rouen, Haute Normandie, France	NCT02021253	Roussel et al. [182].
2. **Prebiotics and diets**						
Colon cancer liver metastasis	250	Oral administered 4 soft gelatin capsules: 1 capsule containing 1 g pure eicosapentaenoic acid (EPA).	NA	Recruiting: University of Leeds	NCT04682665	-
3. **FMT**						
HCC	12	Combination: FMT with Atezolizumab plus Bevacizumab. After a single FMT, patients will continue to receive atezolizumab/bevacizumab every 21-days according to protocol.	NA	Not recruiting yet: Medical University of Vienna	NCT05750030	-
